# Modern Determinants of Earlier Menarche and Mental Health: A Translational Review for Clinicians

**DOI:** 10.3390/children13081068

**Published:** 2026-08-12

**Authors:** Giuseppe Marano, Claudia d’Abate, Giuseppe Sorrenti, Gianandrea Traversi, Osvaldo Mazza, Marianna Mazza

**Affiliations:** 1 Department of Neuroscience, Head-Neck and Chest, Section of Psychiatry, Fondazione Policlinico Universitario Agostino Gemelli IRCCS, Largo Agostino Gemelli 8, 00168 Rome, Italy; 2 Department of Neuroscience, Section of Psychiatry, Università Cattolica del Sacro Cuore, 00168 Rome, Italy; 3 Department of Gynecology, San Carlo di Nancy Hospital, 00165 Rome, Italy; 4 Unit of Medical Genetics, Department of Laboratory Medicine, Ospedale Isola Tiberina-Gemelli Isola, 00186 Rome, Italy; gianandrea.traversi@gmail.com; 5 Spine Surgery Department, Bambino Gesù Children’s Hospital IRCCS, 00168 Rome, Italy; osvaldo.mazza1973@hotmail.it

**Keywords:** early menarche, pubertal timing, adolescent mental health, endocrine-disrupting chemicals, childhood obesity, self-harm, body image

## Abstract

**Highlights:**

**What are the main findings?**

- The global trend toward earlier menarche is associated with multiple interacting factors, including increased childhood adiposity, exposure to endocrine-disrupting chemicals (EDCs), and early-life psychosocial stress.
- Earlier menarche is associated with an increased risk of psychiatric outcomes, including major depression, anxiety, non-suicidal self-injury, and subclinical disordered eating, potentially linked to a neurobiological mismatch between affective and regulatory brain systems.

**What are the implication s of the main findings?**

- Earlier menarche may be considered a biopsychosocial sentinel event, helping clinicians identify adolescents at higher risk for mental health difficulties.
- Clinical practice may benefit from integrating systematic mental health screening, body-image interventions, and anticipatory guidance into routine pediatric and gynecological care to reduce downstream psychopathology.

**Abstract:**

**Background:** Over the past decades, the global onset of menarche has progressively advanced, driven by complex interactions between metabolic, psychosocial, and environmental factors. Beyond its reproductive significance, menarche represents a critical developmental milestone that coincides with increased vulnerability to mental health difficulties. Contemporary determinants such as childhood obesity, early-life adversity, and exposure to endocrine-disrupting chemicals (EDCs) may accelerate pubertal timing while simultaneously shaping adverse psychological trajectories during adolescence. **Objectives:** This review aims to provide a translational and clinically oriented synthesis of modern determinants of earlier menarche and their associations with mental health outcomes in girls and adolescents, highlighting implications for early identification, prevention, and integrated clinical care. **Methods:** We conducted a narrative review mapping a scoping literature search conducted using PubMed/Medline, Scopus, and PsycINFO, focusing on literature from the last decade (2016–2026), with emphasis on systematic reviews, meta-analyses, and longitudinal cohort studies. Evidence was synthesized across biological, psychosocial, and environmental domains. **Results:** Accumulating evidence indicates that higher childhood adiposity, psychosocial stress, and EDC exposure are consistently associated with advanced pubertal timing. Earlier menarche correlates with an elevated risk of depressive symptoms, anxiety, self-harm, and body image-related distress, although effect sizes vary across populations. Emerging longitudinal data suggest that the peri-menarcheal period represents a critical window for symptom exacerbation. These associations appear to be mediated by biological mechanisms (e.g., hormonal shifts, hypothalamic–pituitary–adrenal (HPA) axis reactivity) and social processes including peer-comparison dynamics and premature sexualization. **Conclusions:** Earlier menarche acts as a biopsychosocial sentinel event rather than an isolated gynecological milestone. Integrating mental health screening and anticipatory guidance into pediatric and gynecological care is essential for early risk detection. A multidisciplinary, equity-oriented approach is required to address both individual vulnerability and broader environmental determinants.

## 1. Introduction

Female puberty encompasses a series of fundamental physiological events, including the appearance of breast budding (thelarche), peak height velocity (PHV), and menarche, which generally occurs approximately 6 months after PHV. The age at menarche varies widely, regulated by an interplay of genetic background and environmental cues. Historically, twentieth-century epidemiological studies identified several factors associated with pubertal onset, including mean annual temperature, physical activity, socio-educational status, housing conditions, family size, and general health [1].

During the twentieth century, substantial improvements in socioeconomic conditions and public health in industrialized countries contributed to a secular trend toward earlier puberty. Historical estimates indicate that the average age at menarche declined by approximately 3 months per decade during this period [2].

Despite these established historical triggers, modern pubertal acceleration cannot be attributed to a single causal factor. Rather, contemporary pubertal timing results from the dynamic, integrated interaction among biological, metabolic, environmental, and psychosocial shifts that characterize modern pediatric populations.

## 2. Materials and Methods

### 2.1. Literature Search Strategy

A structured literature search was conducted to identify studies examining the modern determinants of earlier menarche and their associations with adolescent mental health outcomes. The search was performed across PubMed/MEDLINE, Scopus, and PsycINFO. To capture contemporary secular trends, the window was limited to articles published between January 2016 and May 2026. The literature search was restricted to peer-reviewed articles published in the English language.

The search strategy combined Medical Subject Headings (MeSH) terms and free-text keywords using Boolean operators (AND/OR). The search strings integrated terms for pubertal exposure (“menarche”, “pubertal timing”, “precocious puberty”) with modern determinants (“endocrine disrupting chemicals”, “obesity”, “body mass index”, “family structure”, “nutrition”, “dietary protein”, “physical activity”) and psychological outcomes] (“mental health”, “depression”, “anxiety”, “self-harm”, “body image”, “distress”, “psychopathology”). Reference lists of retrieved systematic reviews and meta-analyses were manually screened to identify additional relevant literature.

To align with rigorous scoping review standards, our core research question was structured using the PIPoH framework as follows:

Population (P): Female children, adolescents, and transitional age youth.

Intervention/Exposure (I): Modern biological, metabolic, environmental, and psychosocial determinants (e.g., elevated BMI, EDCs, nutritional shifts, lifestyle factors, stress).

Profession/Context (P): Pediatricians, gynecologists, child psychiatrists, and allied adolescent healthcare professionals.

Outcome (O): Advanced pubertal timing/earlier menarche and associated adolescent mental health outcomes (internalizing disorders, NSSI, disordered eating).

Healthcare Setting (H): Primary pediatric care, adolescent gynecology clinics, and mental health settings.

### 2.2. Eligibility Criteria

Studies were eligible if they evaluated female children, adolescents, or transitional age youth for pubertal timing or age at menarche. Included papers had to investigate contemporary biological, metabolic, psychosocial, or environmental determinants (e.g., BMI, EDCs, dietary patterns, family dynamics) and explicitly report on associated mental health outcomes or emotional distress. The review focused on original peer-reviewed clinical studies—including longitudinal cohorts, randomized controlled trials, and case–control designs—as well as systematic reviews and meta-analyses.

Exclusion criteria comprised case reports, small case series, conference abstracts, editorials, letters, and non-peer-reviewed literature. Preclinical or animal model studies were excluded unless they provided direct translational insights into the biological or epigenetic mechanisms regulating human pubertal timing.

### 2.3. Study Selection and Data Extraction

Titles and abstracts were independently screened by the authors against the predefined selection criteria, and full-text articles of potentially relevant studies were retrieved for comprehensive assessment. Discrepancies were resolved through consensus.

Data extraction focused on study design, population demographics (sample size, geographical region, ethnicity), specific pubertal markers (age at menarche, PHV, or breast development), evaluated determinants, and reported psychiatric outcomes. We specifically extracted variables evaluating mediation or moderation within the peri-menarcheal window.

The selection process was conducted in accordance with PRISMA-ScR (Preferred Reporting Items for Systematic Reviews and Meta-Analyses Extension for Scoping Reviews) guidelines. A total of 842 records were identified through database searches (PubMed/MEDLINE: 340; Scopus: 295; PsycINFO: 207). After removing duplicates (n = 215), 627 unique titles and abstracts were screened independently by two reviewers. Of these, 509 records were excluded as non-relevant. The remaining 118 full-text articles were evaluated for eligibility, leading to the exclusion of 29 articles (14 lacked explicit mental health outcomes; 15 were brief commentaries/editorials). Ultimately, 89 studies met all inclusion criteria and were included in the final qualitative synthesis.

### 2.4. Narrative Synthesis and Conceptual Framework

Due to marked heterogeneity in study designs, population demographics, dietary assessment methodologies, and psychometric instruments, a quantitative meta-analysis was not feasible. Instead, the evidence was integrated using a narrative thematic synthesis. The literature was organized into predefined thematic domains mapping to the core determinants and clinical outcomes: EDCs and epigenetic modulations; BMI, hyperinsulinemia, and metabolic pathways; family environments and psychosocial stress; prepubertal macronutrient intake; and the confounding or protective role of physical activity.

**Figure 1:**
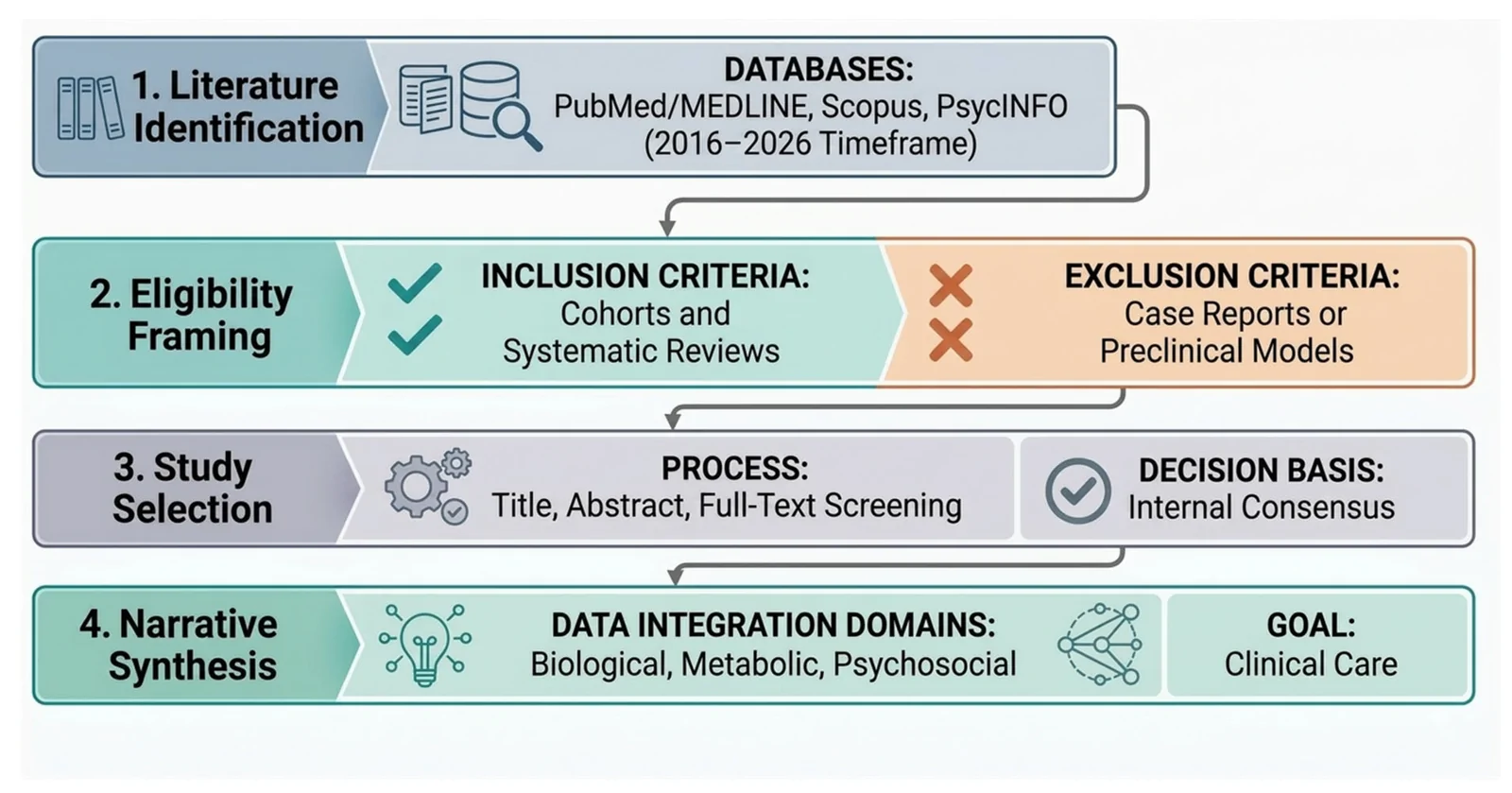
Conceptual flow of literature selection and narrative synthesis for pubertal timing and mental health outcomes. Note: This flowchart illustrates the structured, four-stage methodological process used to identify, select, and narratively synthesize the evidence for this review. The diagram maps the sequential phases of literature identification across PubMed/MEDLINE, Scopus, and PsycINFO within the 2016-2026 timeframe, eligibility framing utilizing strict criteria focused on original cohorts and systematic reviews, study selection via rigorous abstract and full-text screening, and narrative synthesis, where heterogeneous data are integrated into biological, environmental, and psychosocial domains to form a clinically actionable framework for pediatric and gynecological care.

This synthesis bridges the gap between biological vulnerability and clinical pediatric practice, providing a translational framework for integrated clinical care. The operational workflow of literature identification, screening, and multi-domain integration is illustrated in Figure 1.

## 3. Physiology

The ontogeny of female reproductive maturation is governed by the tightly orchestrated activation, silencing, and reactivation of the hypothalamic–pituitary–gonadal (HPG) axis. This developmental trajectory initiates during fetal life through the establishment of the pulsatile gonadotropin-releasing hormone (GnRH) pulse generator, culminating in reproductive competence post-puberty [3].

Hypothalamic GnRH is secreted into the hypophyseal portal circulation to drive the periodic release of luteinizing hormone (LH) and follicle-stimulating hormone (FSH) from the anterior pituitary. In females, FSH stimulates ovarian granulosa cells, promoting follicular maturation and the synthesis of estrogens and inhibin. Conversely, LH tracks at low tonic levels during childhood, shifting to high-amplitude pulses peri-pubertally to trigger thecal androgen production and synchronize the ovulatory cycles that define mature gynecological function [4].

From a chronological perspective, the HPG axis exhibits distinct windows of activity and quiescence that are highly sensitive to modern environmental and metabolic disruptors. Mid-gestational fetal life exhibits high gonadotropin activity, which subsequently declines due to central inhibitory mechanisms and maternal estrogen negative feedback. Following birth, the withdrawal of placental steroids induces a transient neuroendocrine reactivation—termed “mini-puberty”—characterized by elevated serum LH and FSH levels that can persist for over six months. Thereafter, the axis enters a prolonged state of childhood quiescence. During this juvenile pause, basal gonadotropin concentrations remain profoundly suppressed due to an exquisite sensitivity of the hypothalamus to the negative feedback of low-level circulating steroids, alongside strong central GABAergic and neuropeptide Y (NPY) inhibition [4].

The escape from this childhood inhibition marks the peripubertal transition. Endogenous GnRH secretion increases in both frequency and amplitude, initially manifesting exclusively during the early hours of sleep in tandem with nocturnal growth hormone surges. As pubertal maturation advances, these pulsatile peaks extend into daytime hours, eventually dismantling the childhood circadian variation to establish mature adult profiles [3].

Crucially, the molecular architecture driving this reactivation centers on upstream epigenetic and gating networks rather than changes in GnRH synthesis itself, as hypothalamic GnRH content remains relatively static across development [4]. A pivotal gatekeeper of this cascade is kisspeptin, encoded by the KISS1 gene, which binds to the G-protein coupled receptor GPR54 (KISS1R) on GnRH neurons. Hypothalamic kisspeptin signaling increases significantly during the peripubertal window, serving as the primary obligate trigger for pulsatile GnRH release. The clinical weight of this pathway is underscored by human genetic models: loss-of-function mutations in GPR54 result in idiopathic hypogonadotropic hypogonadism, whereas gain-of-function variants or hyper-expression drive central precocious puberty [4].

Emerging evidence further links this neuroendocrine gatekeeper to systemic metabolic and environmental inputs. The kisspeptin network operates as a metabolic sensor integrated with circadian molecular clocks, notably through transcriptional regulators like CLOCK and BMAL1. This integration is highly clinically relevant: disruptions in peripheral metabolic signaling (such as hyperinsulinemia or hyperleptinemia) and central circadian mismatch can directly dysregulate the KISS1/KISS1R system. This provides a clear neurobiological mechanism whereby modern lifestyle shifts bypass standard physiological timelines, accelerating GnRH pulsatility and precipitating earlier menarche.

**Figure 2:**
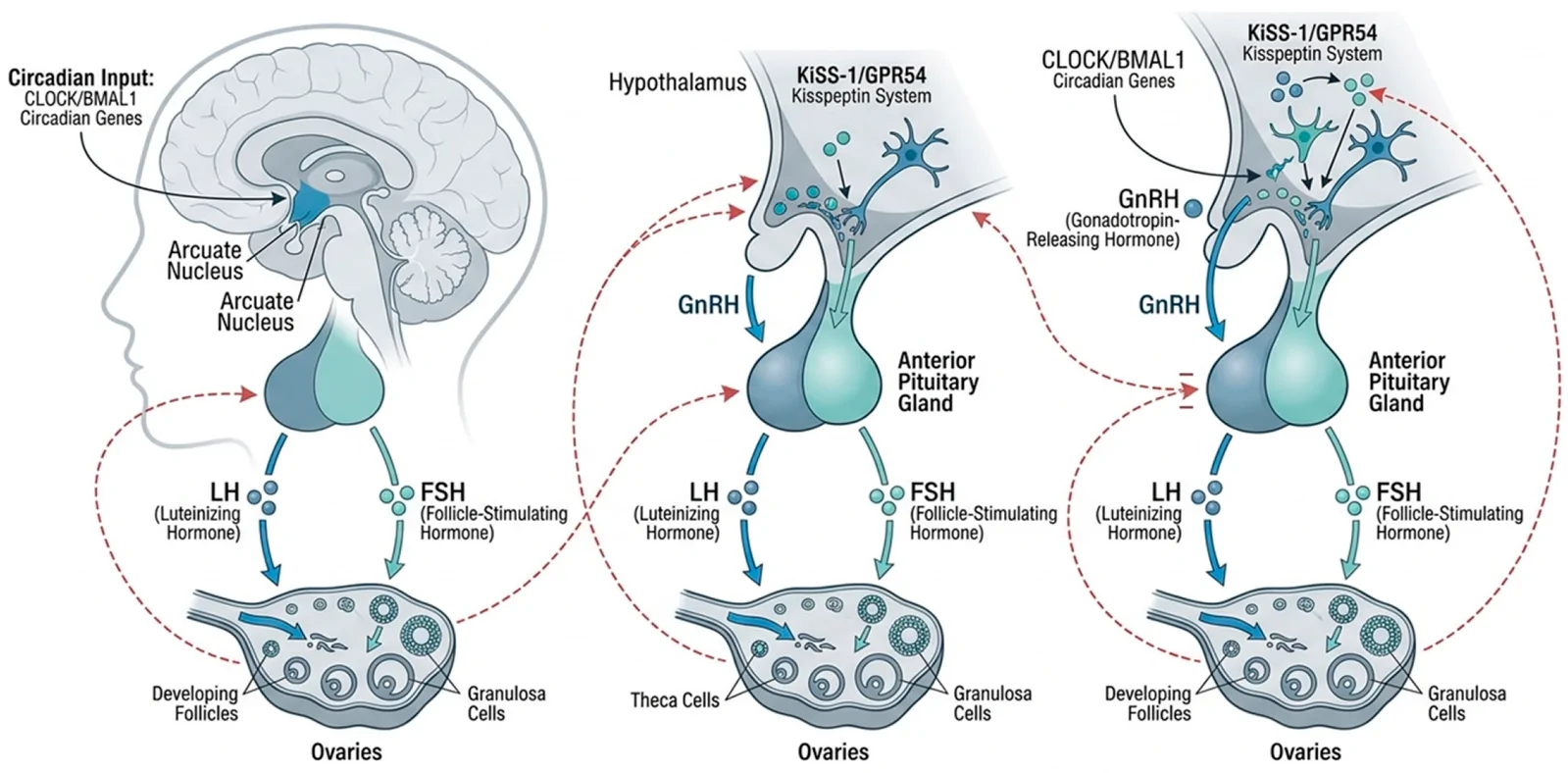
Neuroendocrine regulation of female puberty and the hypothalamic–pituitary–gonadal (HPG) axis. Note: The diagram illustrates the pulsatile release of GnRH from the arcuate nucleus of the hypothalamus, stimulating the anterior pituitary to periodically secrete LH and FSH. The system is modulated by peripheral feedback from ovarian estrogens and inhibin, alongside central molecular regulators including the KiSS-1/KISS1R system, and circadian clock genes (CLOCK and BMAL1). Up-regulation or loss-of-function mutations within these pathways shift the metabolic and temporal gating of pubertal onset.

The functional components of the HPG axis, alongside these upstream molecular, metabolic, and circadian regulators, are schematically integrated and illustrated in Figure 2.

## 4. Modern Determinants of Earlier Menarche

Over the years, numerous factors potentially involved in the development of early menarche have been investigated.

When evaluating contemporary drivers of pubertal timing, it is paramount to note that the vast majority of human literature relies on observational, cross-sectional, or prospective cohort designs. Consequently, while robust statistical associations have been identified, these data reflect correlational patterns rather than established direct causality.

### 4.1. Endocrine-Disrupting Chemicals

Over the last two decades, the impact of endocrine-disrupting chemicals (EDCs) on the female reproductive axis has emerged as a key research priority. These exogenous compounds or mixtures interfere with any aspect of hormone action [5], encompassing structurally diverse classes such as phthalates, phenols, phytoestrogens, organochlorine pesticides, polybrominated flame retardants, diphenyl ethers, and perfluorinated compounds.

However, epidemiologic data linking EDC exposure to advanced pubertal timing remain inconsistent and often contradictory [6,7]. This lack of consensus stems from structural methodological challenges rather than a lack of biological plausibility. Because these compounds exhibit highly variable pharmacokinetic profiles—ranging from short half-lives of a few hours (e.g., phthalates, bisphenol A) to persistent bioaccumulation over decades (e.g., perfluorinated compounds and organochlorines)—cross-sectional biomonitoring is inherently flawed. The choice of biological matrix (urine for non-persistent chemicals versus serum or hair for persistent compounds) and the developmental timing of sampling relative to critical windows of hypothalamic sensitivity significantly confound downstream associations [8].

Despite these limitations, translational evidence supports a direct role for EDCs in accelerating pubertal cascades. In vivo models demonstrate that EDCs induce advanced pubertal onset via peripheral estrogenic mimicking and central epigenetic remodeling, specifically through altered DNA methylation and histone acetylation within the arcuate nucleus kisspeptin (KISS1) promoters [9]. This environmental acceleration is starkly evident in specific demographic subsets. For instance, a classic epidemiological sentinel case involved foreign-born migrating children in Belgium, who exhibited an incidence of central precocious puberty approximately 80-fold higher than native-born counterparts. This surge was strongly correlated with elevated serum levels of p,p’-DDE (a persistent metabolite of the organochlorine pesticide DDT), illustrating how abrupt changes in environmental exposure profiles can trigger early HPG axis reactivation [10].

Therefore, while translational and animal models demonstrate clear biological plausibility for EDC-induced HPG axis activation, human epidemiological findings remain heterogeneous, and current data do not allow for definitive causal inferences regarding environmental chemical exposure and advanced menarche.

### 4.2. Childhood Adiposity and the Obesity Epidemic

Globally, the secular trend toward earlier menarche has closely mirrored the rising prevalence of childhood obesity and elevated Body Mass Index (BMI) [11,12]. This shifting metabolic landscape is fundamentally driven by contemporary socio-environmental transitions, including unprecedented caloric density and energy availability [13,14], systemic sedentary behaviors, escalated screen time [15], and chronic sleep deprivation, which alters hunger-regulating neuroendocrine pathways [16]. Furthermore, children born small for gestational age (SGA) represent a distinct high-risk cohort; they frequently experience rapid postnatal catch-up growth, leading to premature adrenarche, exaggerated adrenal androgen secretion, and subsequent pubertal acceleration [17].

Elevated BMI drives early pubertal onset through a complex, bidirectional causal loop. Genetic correlation analyses reveal a substantial overlap between the loci regulating metabolic homeostasis and those governing pubertal timing, making it difficult to disentangle whether specific genetic variants primarily modulate the adiposity set-point or directly accelerate the reproductive axis itself [5,18].

Beyond environmental and metabolic factors, constitutional advancement of growth represents a major primary determinant of earlier pubertal timing in healthy girls. This physiological variation often intersects with an early adiposity rebound—the early second rise in Body Mass Index occurring around ages 4 to 6 years—which strongly predicts premature adrenarche, exaggerated adrenal androgen secretion, and subsequent pubertal acceleration [17]. Furthermore, shared genetic architectures directly link early-onset obesity and central pubertal timing. A prominent example is represented by loss-of-function mutations in the Makorin Ring Finger Protein 3 (MKRN3) gene, the most common monogenic cause of familial central precocious puberty. MKRN3 acts as an inhibitory central gatekeeper; its loss of function prematurely disinhibits hypothalamic GnRH secretion while simultaneously being implicated in early metabolic dysregulation and childhood obesity, illustrating the deep genetic co-regulation of metabolic set-points and reproductive maturation [19].

Peripherally, adiposity acts via clear metabolic pathways. Excess adipose tissue increases circulating leptin, which acts as a permissive gating signal at the hypothalamus, and induces hyperinsulinemia. Insulin directly suppresses hepatic synthesis of Sex Hormone-Binding Globulin (SHBG), thereby driving up the free, bioavailable fraction of circulating estradiol. Additionally, hyperinsulinemia stimulates theca cell and adrenal androgen hypersecretion. The clinical validity of this metabolic-reproductive link is confirmed by therapeutic interventions: the administration of insulin-sensitizing agents (e.g., metformin) in pre-pubertal girls with hyperinsulinemia successfully reduces androgen loads and retards rapid, premature pubertal progression [20].

### 4.3. Family Dynamics and Psychosocial Stress

Beyond metabolic and chemical inputs, the HPG axis acts as a transducer of early-life psychosocial stress. Several longitudinal studies have established a robust correlation between adverse childhood experiences, dysfunctional family environments, and accelerated female pubertal maturation [21,22]. A large prospective cohort by Aghaee demonstrated that early childhood exposure to a non-intact family structure significantly increased the risk of precocious pubertal transitions [23].

Critically, the interaction between psychosocial stress and advanced pubertal timing is strictly bidirectional. Early-life adversity and chronic family stress hyper-activate the HPA axis, where elevated corticotropin-releasing hormone (CRH) prematurely uncouples central inhibitory gating, triggering early GnRH pulsatility and advanced menarche. Crucially, the phenotypic presentation of early menarche itself acts as an acute, secondary psychosocial stressor. The sudden somatic divergence from the peer group forces the child into a state of hyper-visibility, exposing her to peer victimization, relational stress, and body dissatisfaction. This secondary distress further reinforces HPA-axis dysregulation, creating a bidirectional feedback loop where stress accelerates biological maturation, and accelerated maturation profoundly amplifies psychological stress.

### 4.4. Nutrition and Macronutrient Intake

Prospective investigations into childhood nutritional architecture—specifically macronutrient composition—highlight diet as an adiposity-independent regulator of pubertal timing [24]. Historically, this literature has been undermined by critical methodological shortcomings, such as relying on isolated self-reported food frequency questionnaires (FFQs) or retrospective menarcheal recall, which introduce profound recall bias and measurement errors. Few studies have utilized objective circulatory biomarkers (e.g., plasma phospholipid fatty acid profiles) or tracked pubertal milestones precisely via longitudinal assessments of peak height velocity (PHV). Furthermore, assessing dietary intake *during* the pubertal transition introduces a high risk of reverse causality, as ongoing hormonal shifts themselves modify appetite and eating behaviors [5].

To circumvent these biases, recent analyses have focused on early prepubertal dietary windows. While older data from the UK Avon Longitudinal Study of Parents and Children (ALSPAC) [25] and Canadian cohorts [26] linked high total caloric intake at age 10 to earlier menarche, more robust longitudinal data from the ALSPAC cohort using repeated dietary tracking demonstrated that elevated energy intake during early childhood (ages 3 to 7.5 years) predicts advanced breast development, earlier PHV, and earlier menarche, completely independent of the child’s actual adiposity status during puberty [27]. This suggests that early-life caloric abundance serves as a primary, direct signal to the central pubertal gatekeepers.

Beyond direct macro- and micronutrient signaling, the complex interactions of dietary architectures dynamically reshape the host intestinal ecosystem, positioning the gut microbiota as a critical metabolic and endocrine transducer of pubertal onset.

The gut microbiota plays a primary biological role in regulating homeostasis and the general health of the female organism. In particular, recent evidence has redefined the understanding of the microbiota as a central element for maintaining both physical and psychological well-being in women [28]. The fluctuations and specific composition of the female gut bacterial flora represent a true pillar for the systemic stability of the organism, influencing metabolic, immune, and hormonal pathways closely connected to gender physiology [28]. Parallelly, alterations in the bacterial flora, or dysbiosis, can significantly correlate with neuroendocrine dysfunctions and affective disorders, outlining new challenges and promising opportunities in the field of mood disorder research [29].

Pubertal transition and the timing of menarche are traditionally described as processes governed by the activation of the hypothalamus–pituitary–gonadal (HPG) axis under the strict control of genetic and neuroendocrine factors. However, in recent years, the classical paradigm has been joined by the concept of the gut–brain–gonadal axis, demonstrating how signals originating from the gastrointestinal tract and mediated by microorganisms are capable of profoundly modulating sexual maturation. Central precocious puberty (CPP) in girls, characterized by the premature activation of the HPG axis and the development of secondary sexual characteristics before the age of 8, represents an ideal clinical model to study these interactions [30]. This phenomenon leads to accelerated bone maturation and compromises final height, while also increasing the adult risk of cardiovascular diseases, type 2 diabetes, and estrogen-dependent tumors [30].

Both clinical studies in humans and experimental models in animals indicate that alterations in the microbiota and its derived metabolites actively participate in the progression of precocious puberty [30,31]. During normal puberty, the microbiota of girls undergoes a remodeling driven by sex hormones, with an increase in the *Firmicutes phylum*, particularly the *Lachnospiraceae* and *Ruminococcaceae families*, and an enrichment of the *Clostridiales* order at the expense of *Bacteroidales* [30]. In girls affected by CPP, a profound alteration of this trajectory is found instead, often characterized by an increase in alpha-diversity and an imbalance in the *Firmicutes*/*Bacteroidetes* ratio [30].

The estrobolome plays a major role in this context, comprising the collection of gut bacteria belonging to genera such as *Bacteroides*, *Clostridium*, *Escherichia*, and *Lactobacillus*, equipped with the beta-glucuronidase enzyme. This enzyme can deconjugate estrogens that were conjugated by the liver and excreted in the bile, allowing their trans-epithelial reabsorption and re-entry into the systemic circulation through the enterohepatic circulation. In patients with CPP, the enrichment of taxa like *Escherichia* and *Klebsiella* determines an increase in fecal beta-glucuronidase activity, prematurely amplifying the amount of bioavailable estrogens and accelerating pubertal development [30].

Additionally, short-chain fatty acids (SCFAs), including acetate, propionate, and butyrate, derive from the bacterial fermentation of dietary carbohydrates. In many cohorts of CPP patients, a significant depletion of beneficial and butyrate/propionate-producing genera, such as *Bacteroides*, *Faecalibacterium*, and *Anaerostipes*, is observed, with a consequent reduction in fecal SCFAs that is inversely correlated with LH, FSH, and estradiol levels. For example, a propionate deficit reduces the inhibitory stimulus on ghrelin secretion and impairs the release of satiety hormones like PYY and GLP-1, creating a metabolic-neuroendocrine profile favorable to the early activation of GnRH neurons [30].

The causal nature of these interactions is validated by studies on rodents, where high-fat diet-induced dysbiosis accelerates pubertal onset through a direct microbiota-metabolites-KISS1 signaling pathway. The expansion of specific taxa, such as *Lactobacillus* and *Romboutsia*, promotes the production of metabolites like 23-nordeoxycholic acid and psoralidin, which directly stimulate hypothalamic KISS1 expression, triggering GnRH release and the gonadotropin cascade. Targeted antibiotic treatments or microbiota reconstitution can reverse this process, reducing KISS1 and GnRH expression and delaying pubertal activation [30]. Regarding the boundaries with pediatric obesity, although the CPP microbiota shares some characteristics with obesity, such as an elevated *Firmicutes*/*Bacteroidetes* ratio, it is distinguished by a more marked endocrine-specific signature closely coupled with gonadotropin pulsatility and estrobolome hyperactivity, configuring itself as a potential independent biomarker for clinical risk stratification [30].

### 4.5. Specific Macronutrient Signals

Nutritional signaling is highly specific to macronutrient quality. Longitudinal data from the UK (ALSPAC) [25,27] and Germany (DONALD study) [32] confirmed that high total protein intake during prepubertal years significantly accelerates the pubertal cascade. Specifically, the consumption of animal protein relative to plant-derived protein sources is consistently linked to advanced menarche across geographically diverse cohorts in the US [33], Germany [32], and Iran [34]. Mechanistically, rich animal proteins provide a high concentration of essential branched-chain amino acids, which directly upregulate the mechanistic target of rapamycin (mTOR) pathway in the hypothalamus, stimulating KISS1 expression and triggering early GnRH pulsatility.

Regarding dietary lipids, elevated consumption of polyunsaturated fatty acids (PUFAs), particularly n-3 PUFAs, and monounsaturated fatty acids (MUFAs) such as oleic acid, correlates with earlier menarche [5]. Conversely, carbohydrate intake shows divergent trends. While most European cohorts found no significant effect [27,35], a US cohort reported that girls in the highest carbohydrate intake quartile experienced menarche approximately seven months later than those in the lowest quartile [36], potentially reflecting the protective, stabilizing effect of low-glycemic, fiber-rich complex carbohydrates against insulin surges.

### 4.6. The Modulatory Role of Physical Activity

The relationship between childhood physical activity and pubertal timing exhibits marked divergence between observational and prospective designs. A recent systematic review of historical data indicated that prolonged physical activity [37,38,39] and high-intensity exercise [40] are associated with delayed menarche, with physically active girls and athletes experiencing menarche an average of 1.13 years later than non-athletes [41]. This delay has traditionally been attributed to an energy-availability crisis, where high caloric expenditure creates a relative metabolic deficit that suppresses pulsatile GnRH release.

Conversely, the majority of modern prospective, population-based cohort studies report no significant association between standard recreational physical activity levels and pubertal onset after adjusting for baseline BMI [42,43,44]. This discrepancy indicates that while extreme, elite-level athletic training can physically delay the HPG axis via energy deprivation, normal variations in recreational childhood physical activity are insufficient to counteract the dominant, compounding acceleration driven by modern obesogenic and chemical factors.

**Figure 3:**
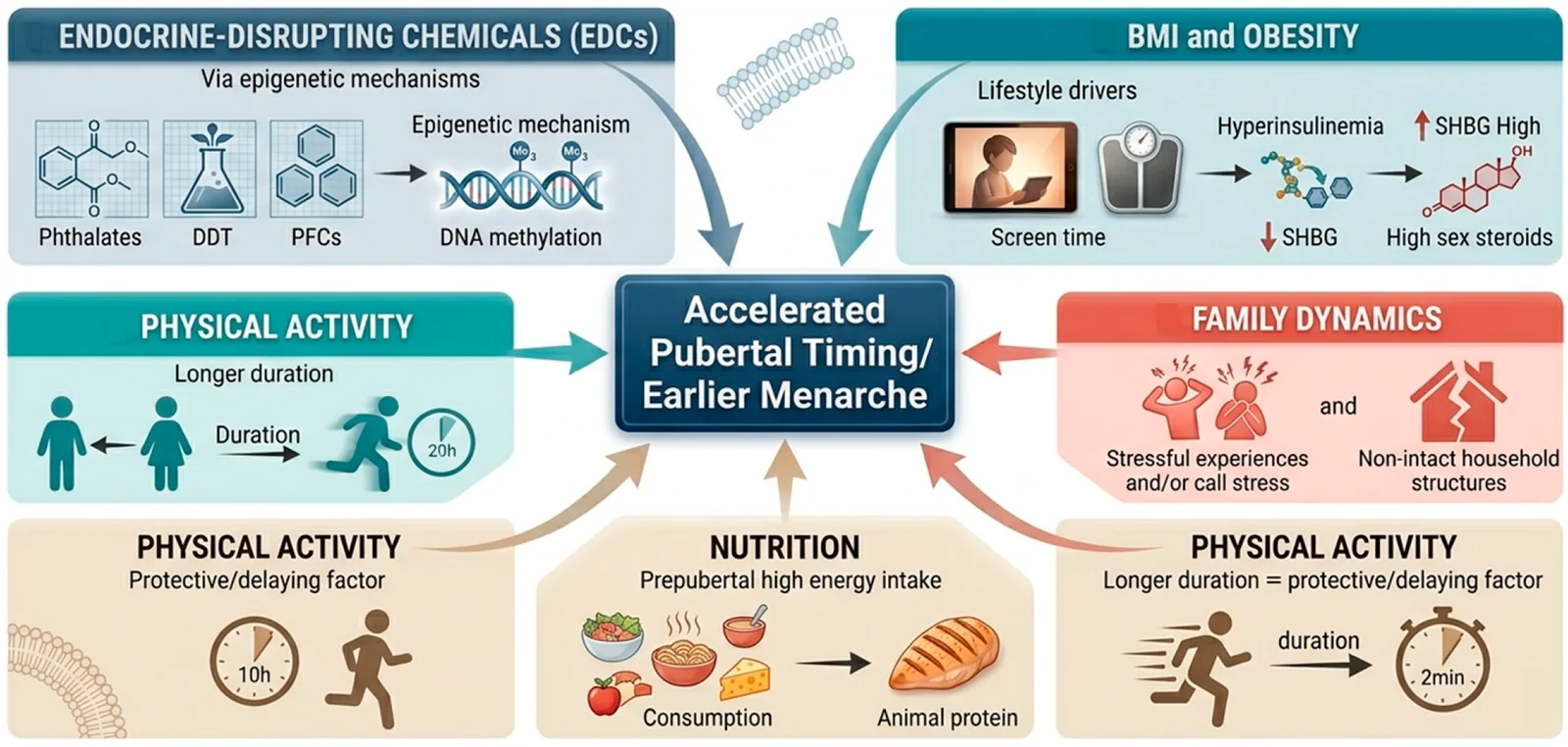
Multifactorial determinants of accelerated pubertal timing and early menarche. Note: Schematic overview of the modern environmental, metabolic, and psychosocial drivers accelerating female pubertal development. Key determinants include endocrine-disrupting chemicals acting via epigenetic mechanisms, elevated childhood BMI driven by hyperinsulinemia and reduced sex hormone-binding globulin (SHBG), stressful family dynamics, high prepubertal animal protein intake, and sedentary lifestyles. These factors interact dynamically to shift the distribution of pubertal onset age.

In summary, the secular trend toward accelerated female puberty is an ongoing, cumulative phenomenon. It cannot be solved by looking at isolated variables; rather, it represents a syndemic where childhood obesity, EDC exposure acting directly on hypothalamic promoters or indirectly via adipose storage, early-life stress-induced HPA axis activation, and specific amino acid dietary signals converge. This multi-layered environmental and metabolic cross-talk is systematically conceptualized and mapped in Figure 3.

### 4.7. Organic and Endocrine Etiologies of Advanced Pubertal Timing

While modern lifestyle and environmental factors drive the broad secular trend toward earlier menarche, clinicians must critically differentiate these from specific, organic endocrine system diseases causing precocious puberty. Central precocious puberty (CPP) can arise from intracranial lesions and tumors affecting the central nervous system, such as hypothalamic hamartomas, optic pathway gliomas, astrocytomas, or congenital arachnoid cysts, which disrupt central inhibitory pathways on the HPG axis [45].

Conversely, peripheral precocious puberty (PPP)—or gonadotropin-independent precocious puberty—can be triggered by autonomous steroid secretion from ovarian cysts, granulosa cell tumors of the ovary, Leydig/Sertoli cell tumors, hormone-secreting adrenal tumors, or genetic conditions like McCune–Albright syndrome. Identifying these organic etiologies is a clinical imperative, as they bypass environmental modulators and require immediate, targeted medical or surgical interventions to arrest accelerated somatic maturation and treat the underlying pathology [46].

### 4.8. Digital Media Exposure and Sleep Deprivation as Neuroendocrine Disruptors

Beyond classical metabolic variables, modern digital lifestyle shifts—specifically escalated screen time and chronic sleep deprivation—have emerged as crucial upstream drivers of accelerated pubertal timing [47].

Prolonged evening exposure to blue light emitted by digital devices suppresses the nocturnal secretion of melatonin from the pineal gland. Because melatonin exerts a tonically inhibitory effect on the GnRH pulse generator during childhood, its suppression accelerates the reactivation of the HPG axis [48]. Furthermore, media-induced sleep restriction alters hunger-regulating pathways by decreasing leptin sensitivity and increasing ghrelin, reinforcing the obesogenic loop that triggers early menarche. Thus, digital media and sleep fragmentation represent key behavioral catalysts that bypass classical physiological timelines [49].

## 5. Internalizing Outcomes

Earlier pubertal maturation is strongly linked to an elevated risk of psychiatric morbidity during adolescence, establishing a distinct pathological trajectory compared to normative or late developmental timelines. Specifically, the phenotypical expression of early menarche operates as a potent amplifier for specific diagnostic clusters, predominantly encompassing fear-related conditions, distress disorders, and externalizing behavioral pathology [50].

When examining the neurobiological underpinnings of early pubertal maturation—such as structural corticostriatal mismatches, altered frontolimbic connectivity, and sensitivity to gonadal steroids—it is critical to distinguish between well-established clinical outcomes and emerging neuroimaging hypotheses. While the link between early menarche and adolescent internalizing symptoms is robustly supported by longitudinal epidemiological data, the underlying neural circuit models are primarily derived from cross-sectional neuroimaging sub-studies. Consequently, these mechanistic pathways represent promising hypothetical models that warrant further validation through prospective, multimodal neuroimaging cohorts rather than fully established pathophysiological certainty.

In a seminal longitudinal cohort tracking 496 adolescents, Stice et al. [51] demonstrated that early-maturing girls exhibited a significantly heightened hazard ratio for major depression and multi-substance abuse—spanning alcohol, tobacco, marijuana, stimulants, inhalants, and hallucinogens—relative to their on-time peers. Paradoxically, this vulnerability matrix appears highly domain-specific, as the same cohort demonstrated no statistically significant escalation in the incidence of core eating disorders, including anorexia nervosa, bulimia nervosa, and binge-eating disorder [52]. This discrepancy underscores that early menarche selectively targets specific neurobehavioral circuits rather than inducing a generalized psychosocial vulnerability. To provide a robust methodological overview and facilitate a structured comparison across the diverse evidence base, the core empirical studies, clinical cohorts, and literature syntheses spanning from reference [49,50,51,52,53,54,55,56,57,58,59,60,61,62,63,64,65,66,67,68,69,70,71,72,73,74,75,76,77,78,79,80,81,82,83,84,85,86,87] are systematically summarized in Table 1.

As systematically mapped in Table 1, the pronounced heterogeneity in clinical and epidemiological findings underscores the necessity of exploring the shared and distinct pathways linking pubertal maturation to psychopathology. Mechanistically, this psychopathological burden is driven by a profound neurobiological mismatch. Gonadal steroid surges during accelerated puberty enhance the neuroplasticity of cortical and subcortical circuits regulating cognitive–emotional processing, opening a dual window for adaptive developmental learning and extreme psychological vulnerability [52]. During adolescence, profound structural and functional remodeling occurs within the prefrontal cortex (PFC) and limbic architecture, including the amygdala and ventral striatum [50,52,53].

This pubertal remodeling is driven by a tightly orchestrated interplay between the organizational and activational effects of circulating ovarian steroids [54,55]. Organizational exposures permanently restructure micro-circuit topography, while activational shifts temporarily modulate neural response thresholds governing reproductive drives, salience detection, motivational salience, and social valuation [55]. These structural alterations are mediated at the cellular level by sex-specific waves of neurogenesis, synaptogenesis, targeted apoptosis, localized synaptic pruning, and progressive myelination [56].

In early-maturing girls, this neurodevelopmental cascade encounters a severe chronological mismatch: the hormone-driven, affect-heavy limbic structures (such as the ventral striatum and amygdala) mature at an accelerated pace, whereas the top-down inhibitory control networks of the prefrontal cortex develop according to strict chronological age. This structural uncoupling severely impairs emotional regulation and impulse control, offering a clear neurobiological explanation for the sudden elevation in depressive phenotypes and reward-seeking substance use seen in this population.

From a psychosocial perspective, this neurobiological vulnerability intersects directly with environmental mismatches. Menarche precipitates the rapid manifestation of secondary sexual characteristics, leading early-maturing girls to be systematically perceived as chronologically older than their peers [57]. This accelerated somatotype promotes premature affiliation with older peer groups, expanding exposure to—and social pressure for—deviant and high-risk behaviors, including early alcohol and substance experimentation [58,59].

Within the internalizing spectrum, depression and anxiety remain the most prevalent clinical manifestations in adolescent girls experiencing advanced pubertal timing [60]. This affective burden is aggravated by an intense concentration of psychosocial stressors, characterized by heightened interpersonal friction, peer victimization [61], and a documented, elevated risk of experiencing unwanted sexual attention or contact [50].

These external stressors are further amplified by gender-specific cognitive coping architectures. Adolescent girls exhibit a higher reliance on rumination—the maladaptive, passive focus on symptoms of distress—which prevents effective emotional processing and chronically activates the hypothalamic–pituitary–adrenal (HPA) stress axis [50]. Furthermore, sudden somatic changes such as acne or rapid adipose tissue deposition clash with socio-cultural body ideals, exacerbating negative body image, social anxiety, and relational distress.

Conversely, adjusted survival analyses demonstrate that later pubertal onset operates as a robust protective buffer. Girls experiencing late menarche display a significantly more resilient body-image phenotype, higher baseline self-esteem, and a marked reduction in depressive symptoms, moving within a lower risk profile for major depressive disorder, panic disorder, and social phobia [50].

In conclusion, under normative timelines, the somatic and psychosocial shifts of puberty represent an adaptive evolutionary process; however, when this cascade is desynchronized and brought forward, the structural mismatch between accelerated subcortical brain development and standard chronological social environments creates an optimal substrate for heterogeneous psychopathological outcomes.

## 6. Body Image Dynamics and Theoretical Frameworks of Pubertal Mismatch

Body dissatisfaction—the structural execution of negative cognitions and affects toward one’s physical schema [62]—reaches clinical salience during the adolescent transition, exhibiting a stark female-biased prevalence [63]. This psychological construct acts as a powerful transdiagnostic vulnerability factor, directly driving the onset of restrictive or dysfunctional eating behaviors, unhealthy weight-control practices, and clinical depression [64]. Crucially, the pubertal window operates as the critical developmental node wherein core body image trajectories are permanently consolidated [65]. On average, girls enter puberty earlier than boys, with a mean age of approximately 11 years in females [66].

To explain the psychopathological risks associated with this transition, developmental psychology relies on two primary classical models: the early-timing hypothesis and the off-timing hypothesis [68]. The early-timing (or “developmental readiness”) hypothesis posits that girls who mature significantly ahead of their peers experience a severe internal desynchronization: their rapid somatic and secondary sexual transformation occurs long before cognitive–emotional consolidation is complete, leaving them poorly equipped to cope with the psychosocial demands of a mature phenotype. Conversely, the off-timing (or “developmental deviance”) hypothesis suggests that any statistical deviation from the peer normative mean—whether accelerated or delayed—induces distress due to the psychological burden of feeling “deviant” from the social group [67].

While historical cohorts occasionally supported the off-timing model for specific externalizing behaviors, contemporary empirical data overwhelmingly validate the early-timing hypothesis as the dominant vector for internalizing pathology and eating disorders in females [68]. This female-specific vulnerability is deeply rooted in the dimorphic nature of pubertal somatotypes. While male pubertal maturation drives lean muscle mass deposition—aligning closely with Western socio-cultural ideals of masculinity—female puberty mandates a mandatory, physiological increase in adipose tissue and a widening of the pelvic girdle. In early-maturing girls, this rapid adipose deposition shifts their body composition abruptly away from the pervasive socio-cultural thin ideal, precipitating early and intense body dissatisfaction.

This metabolic–somatic intersection underscores a critical methodological debate regarding the role of baseline adiposity. Several studies tracking the relationship between pubertal timing and body dissatisfaction in girls note that later-maturing adolescents exhibit significantly lower body dissatisfaction and higher self-esteem. However, prospective analyses demonstrate that this protective effect is frequently attenuated or entirely erased once models are adjusted for pre-pubertal Body Mass Index (BMI) [67]. This attenuation indicates that pre-pubertal adiposity acts as a shared, confounding driving force: higher childhood BMI independently accelerates the metabolic activation of the HPG axis (via leptin signaling) while simultaneously placing the girl at a higher baseline risk for body dissatisfaction before puberty even initiates.

Nevertheless, attributing this pathology solely to baseline BMI represents a reductionist error that ignores key psychosocial dynamics. Regarding subjective self-image, early-maturing girls are significantly less likely to perceive themselves as physically attractive and report chronic feelings of alienation and “otherness” compared to peers, an association that remains highly significant even after rigorous adjustment for objective BMI status [67]. This confirms that the subjective experience of early menarche introduces an independent psychological stressor. The visibility of premature breast development and menarche forces these girls into a state of hyper-visibility, exposing them to peer victimization, sexualized bullying, and adult objectification while they still possess the emotional coping architecture of a child.

Rather than viewing these variables in isolation, future clinical frameworks must evaluate pubertal timing through the “accentuation hypothesis,” exploring how early-life vulnerabilities (such as low socioeconomic status or childhood trauma) are magnified by the acute stress of early menarche. From a clinical and preventative standpoint, identifying early-maturing profiles cannot remain a purely gynecological exercise. Early identification of out-of-time maturers within primary pediatric care is essential to deploy targeted, cognitive-behavioral body image interventions. Uncoupling the somatic transition from psychological distress is a vital clinical priority to disrupt the cascade leading from body dissatisfaction to full-blown eating pathology and adolescent depression.

## 7. Self-Harm, Suicidality, and the Neuro-Developmental Matrix

From a public health perspective, self-harm represents a major concern, with growing evidence indicating that rates of this behavior are increasing, particularly among girls [69], and peaking during adolescence [70]. Earlier pubertal timing has been consistently associated with an increased risk of suicide attempts in both cross-sectional [67,72,73] and prospective studies [74]. Specifically, cross-sectional research conducted among Chinese adolescents has shown that girls with earlier pubertal development, assessed through age at menarche, were significantly more likely to engage in self-harming behaviors [75,76]. However, findings in the broader literature remain inconsistent, as several studies have failed to identify evidence supporting this association [77,78]. Part of this heterogeneity may be explained by the widespread use of subjective measures of pubertal timing. Furthermore, among studies investigating this relationship, only a limited number have differentiated between self-harm with and without suicidal intent. Previous longitudinal data have shown that suicidal and non-suicidal self-harm share certain risk factors while also presenting distinct characteristics, suggesting that these behaviors overlap but are not entirely equivalent [79].

From a psychosocial perspective, this biological vulnerability directly intersects with severe environmental mismatches that challenge the individual’s coping capacity. Since early menarche has been associated with depression [80] and depressive symptoms [81]—both of which are among the strongest risk factors for self-harm [82]—there appears to be a complex association linking age at menarche, depression, and self-harm. These conditions may also share common underlying biological mechanisms; indeed, it has been hypothesized that hormones involved in pubertal development may directly influence neural structures, such as the hippocampus and amygdala, through dopaminergic and serotonergic pathways, thereby contributing to depressive symptoms [83]. In fact, substantial neurological changes in these regions have been documented during puberty [52]. The imbalance between the maturation of reward-seeking neural circuits and the later development of the prefrontal cortex, which is involved in executive functioning, has been proposed as a possible explanation for the increase in risk-taking behaviors—including self-harm—observed during adolescence, with a particularly elevated vulnerability among early-maturing individuals [82].

This accelerated somatic development forces a rapid divergence from the normative peer group, triggering negative social comparison processes, body image disturbances, and severe psychosocial stress. These dynamics strongly align with the developmental readiness (or “early timing”) hypothesis, which states that accelerated maturers face the most severe psychopathological consequences because they lack the cognitive and emotional consolidation required to navigate the complex social pressures of a physically mature phenotype [82].

Specifically, the early manifestation of secondary sexual characteristics shifts how these girls are perceived by adults and peers alike, often resulting in their treatment as chronologically older than their actual age [84]. This premature adultification frequently leads to the early initiation of sexual and romantic relationships [60]. Consequently, early-maturing girls are thrust into complex, non-normative interpersonal environments before they have developed the necessary psychological resilience. They encounter sophisticated stressors—such as romantic conflicts, relational victimization, and sexual pressures—that significantly exceed their emotional readiness [85]. Romantic relationship distress, in particular, acts as an acute proximal trigger for non-suicidal self-injury (NSSI) in early-maturing females, who utilize the behavior to manage overwhelming interpersonal anxiety [86]. Conversely, later-maturing girls benefit from a developmental buffer; they are afforded the opportunity to observe and cognitively process pubertal transitions within their peer group before experiencing those physical and social shifts firsthand.

In conclusion, the empirical data reveal a clear inverse relationship between pubertal timing and self-harm trajectories in females. An advanced age at menarche operates as a long-term protective shield, significantly reducing the lifetime risk of self-harm across both adolescence and emerging adulthood. These dynamics reinforce the status of early pubertal timing as a critical, transdiagnostic risk factor that requires early clinical screening and targeted, resilience-building interventions.

## 8. Disordered Eating Frameworks

To accurately evaluate the nutritional vulnerabilities associated with accelerated maturation, a rigorous nosological distinction must be maintained between fully consolidated clinical eating disorders and the broader spectrum of disordered eating (DE). Disordered eating defines a constellation of abnormal eating and maladaptive weight-control behaviors—including restrictive dieting, chronic fasting, binge eating, systematic meal skipping, and self-induced vomiting—that do not fully satisfy the rigid diagnostic thresholds of clinically defined eating disorders, yet represent highly damaging, ineffective strategies for somatic regulation [87]. Despite their sub-clinical classification, these behaviors induce severe physical and psychological morbidity, driving a drastic reduction in adolescent quality of life.

Epidemiological mapping demonstrates that DE phenotypes are profoundly more prevalent than full-blown clinical diagnoses [88], cutting across diverse demographic boundaries, including varied age cohorts, ethnic minorities, and populations within developing nations [87]. The etiology of DE is inherently multifactorial, emerging from a complex intersection of genetic predispositions, neurobiological changes, sociocultural pressures, and localized environmental stressors [86]. Consequently, mapping high-risk trajectories during early development is a clinical imperative for the deployment of targeted preventative protocols.

Prospective and cross-sectional data consistently isolate early pubertal timing as a core catalyst for DE symptomatology, resolving a critical paradox in the literature. While accelerated puberty does not uniformly elevate the risk for highly structured, low-frequency clinical phenotypes like classic Anorexia Nervosa, it acts as a potent, direct driver of sub-clinical DE behaviors [68]. Puberty represents a high-vulnerability developmental window wherein intricate interactions between gonadal steroid surges, rapid somatic shifts, and psychological re-indexing transition the individual from childhood to reproductive maturity. This biological milestone is marked by profound interindividual variance in chronological onset [89].

**Figure 4:**
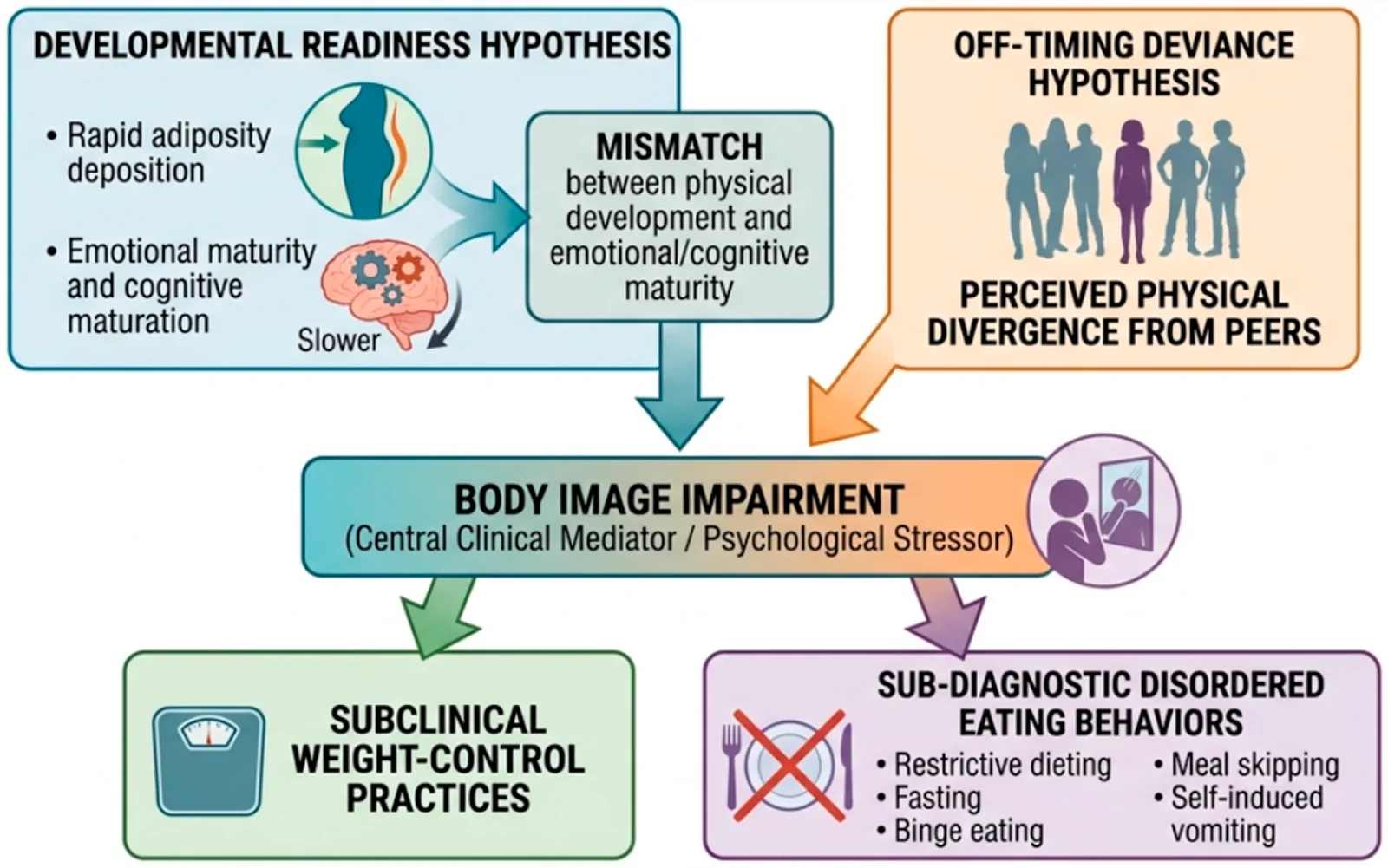
Theoretical model of the psychological mechanisms linking early puberty to dysfunctional eating behaviors. Note. Theoretical model illustrating the main psychological mechanisms through which early puberty and earlier menarche may contribute to body image impairment and maladaptive eating behaviors. The developmental readiness hypothesis proposes that rapid physical maturation is not accompanied by adequate emotional and cognitive development, resulting in a developmental “mismatch.” In parallel, the off-timing deviance hypothesis suggests that perceiving oneself as physically different from peers may act as a significant psychological stressor. Body image impairment therefore emerges as a central clinical mediator associated with both subclinical weight-control practices and sub-diagnostic disordered eating behaviors, including restrictive dieting, fasting, binge eating, meal skipping, and self-induced vomiting.

As previously established, early pubertal timing systematically forces an acute developmental mismatch, wherein accelerated physical and secondary sexual transformation occurs long before cognitive–emotional consolidation is achieved. A comprehensive meta-analysis focusing predominantly on female cohorts confirmed a robust, direct association between accelerated pubertal onset and elevated eating disorder symptomatology during adolescence, validating the hypothesis that early maturation specifically lowers the threshold for DE execution [68]. This vulnerability is heavily driven by the somatic presentation of early menarche: the rapid deposition of adipose tissue directly contradicts rigid Western thinness ideals, generating intense body dissatisfaction that adolescents attempt to manage through restrictive or purgative behaviors (Figure 4).

Earlier age at menarche has also been associated with body dissatisfaction and binge eating among young adult women [87].

However, further studies conducted on larger samples, with appropriate adjustment for potential confounding factors, are still needed in order to provide more comprehensive evidence on this issue.

## 9. Limitations

This review must be interpreted in light of several methodological limitations inherent to the available literature. First, a major challenge in isolating independent determinants of pubertal timing is residual confounding; key variables such as prepubertal BMI, socioeconomic status, household stress, and ethnic background are tightly intertwined, making it difficult to fully isolate individual drivers. Second, many large-scale epidemiological cohorts rely on retrospective self-reported age at menarche or subjective pubertal timing assessments rather than prospective, physician-rated Tanner staging, introducing potential recall and reporting bias. Third, while the association between early menarche and internalizing psychopathology is well-established, downstream neurobiological mechanisms (e.g., corticostriatal structural mismatches) largely represent emerging translational hypotheses derived from neuroimaging sub-samples rather than universally validated clinical biomarkers.

## 10. Conclusions

The contemporary global decline in the age at menarche represents a complex, multifactorial phenomenon shaped by a reciprocal interaction of metabolic, psychosocial, environmental, and lifestyle-related determinants, notably childhood obesity, exposure to endocrine-disrupting chemicals (EDCs), and adverse early-life environments. Beyond its reproductive and gynecological significance, earlier menarche operates as a critical, transdiagnostic clinical marker of neurodevelopmental and psychosocial vulnerability, drastically elevating the risk for internalizing pathology, non-suicidal self-injury (NSSI), and sub-clinical disordered eating behaviors in adolescent girls. These findings mandate a paradigm shift beyond a reductionist gynecological interpretation of pubertal timing in favor of an integrated biopsychosocial framework. Advanced pubertal timing offers primary pediatric and adolescent healthcare providers a highly visible, accessible sentinel indicator of psychosocial risk. From a clinical perspective, these findings do not mandate unvalidated universal screening protocols, but rather suggest a risk-informed approach. In alignment with guidelines from international bodies such as the US Preventive Services Task Force (USPSTF) [90] and the American Academy of Pediatrics (AAP) [91]—which recommend screening for major depressive disorder in adolescents aged 12–18—clinicians should consider early menarche as a key clinical indicator of heightened vulnerability. Incorporating anticipatory guidance, body-image support, and targeted emotional well-being checks during routine pediatric and gynecological visits can help identify early distress and facilitate timely intervention before severe psychopathology consolidates.

## Figures and Tables

**Table 1:** Methodological mapping and clinical synthesis of key empirical studies, longitudinal cohorts, and reviews on pubertal timing and adolescent psychopathology (References [45,46,47,48,49,50,51,52,53,54,55,56,57,58,59,60,61,62,63,64,65,66,67,68,69,70,71,72,73,74,75,76,77,78,79,80,81,82]). Note: This table provides a systematic overview of the literature evaluated within the psychiatric, behavioral, and neurobiological sections of this review. Abbreviations: ALSPAC, Avon Longitudinal Study of Parents and Children; BMI, Body Mass Index; DE, Disordered Eating; EDCs, Endocrine-Disrupting Chemicals; HPG, Hypothalamic–Pituitary–Gonadal axis; NSSI, Non-Suicidal Self-Injury; PHV, Peak Height Velocity. Studies are grouped or chronologically ordered according to their contextual integration within the text to contrast empirical design variations (e.g., cross-sectional vs. prospective cohorts) and highlight specific methodological limitations.

Reference/Study	Study Design	Population/Sample Size	Key Findings	Limitations
Platt et al., 2017 [50]	Latent Modeling Analysis	Large epidemiological sample	Early menarche acts as a transdiagnostic risk factor, predicting broad, non-specific psychiatric disorder vulnerabilities.	Cross-sectional nature of the primary datasets utilized.
Goddings et al., 2014 [52] Ladouceur, 2012 [53] Schulz et al., 2009 [54] Blakemore et al., 2010 [55] Ahmed et al., 2008 [56]	Neuroimaging & Biological Syntheses	Diverse adolescent brain imaging cohorts	Documented pubertal hormone influences on subcortical brain architecture (hippocampus/amygdala). Unveils a mismatch between early reward-seeking maturation and late prefrontal cortex development.	High inter-individual variability; challenging to isolate purely hormonal changes from social context.
Patton et al., 2004 [57] Bachman et al., 2018 [58] Wilson, 1994 [59] Copeland et al., 2010 [60]	Longitudinal & Epidemiological Cohorts	Large-scale youth registries (e.g., Great Smoky Mountains Study)	Accelerated pubertal timing strongly predicts the premature initiation of risk-taking behaviors, including alcohol/tobacco abuse and early substance use trajectories.	Attrition in older waves; shifts in historical drug trends over the longitudinal timeline.
Hankin et al., 2007 [61] Angold & Costello, 2006 [83]	Prospective and Clinical Syntheses	Adolescent community samples	Identifies gender-specific vulnerabilities in depression emergence during puberty, heavily moderated by acute stress exposure and stress reactivity.	Rely heavily on subjective stress scales.
Grogan, 2021 [62] Al Sabbah et al., 2009 [63] Neumark-Sztainer et al., 2006 [64] Reel et al., 2015 [65]	Cross-Sectional Surveys & Longitudinal Cohorts	Multi-national cohorts (up to 24 countries)	Early physical maturation induces severe body image disturbances, weight dissatisfaction, and negative social comparison, which prospectively drive maladaptive health behaviors.	Self-reported weight metrics; cultural differences in body ideals across different nations.
Patton & Viner, 2007 [66] Tarif et al., 2025 [67] Ullsperger & Nikolas, 2017 [68]	Systematic Reviews & Prospective Cohorts	Large birth cohorts (2025 data included) and meta-analytic samples	Confirms the persistent link between early pubertal tempo, negative self-image, and transdiagnostic psychopathology, highlighting marked female vulnerability.	High heterogeneity in pubertal stage measurements across older meta-analyzed data.
Griffin et al., 2018 [69] Gillies et al., 2018 [70]	National Registry Study & Meta-Analysis	Multi-center global samples (10-year registries)	Documented a significant global increase in adolescent self-harm rates, with clinical peaks specifically concentrated within female adolescent demographics.	Registry data may underreport non-suicidal self-harm cases treated outside clinical settings.
Tondo et al., 2017 [71]	Clinical Retrospective Study	Clarified psychiatric patient samples	Early age at menarche significantly predicts an earlier clinical onset of major affective (depressive) and severe anxiety disorders.	Retrospective recall of menarcheal age in adult clinical patients.
Graber et al., 1997 [72] Larsson & Sund, 2008 [73]	Prospective Cohort Surveys	Scandinavian and US school-aged cohorts	Highlighted inconsistencies in the literature; some cohorts failed to find direct paths between timing and long-term self-harm, pointing to methodological differences.	Shorter follow-up windows; variations in how deliberate self-harm was defined.
Wichstrøm, 2000 [74]	National Longitudinal Study	*n* = 2924 Norwegian adolescents	Early pubertal timing acts as a distinct predictor for adolescent suicide attempts, detailing overlapping yet non-equivalent risk paths with non-suicidal self-harm.	Measured puberty via perceived/subjective timing instead of strict clinical or biological markers.
Chen et al., 2017 [75] Deng et al., 2011 [76] Liu et al., 2018 [78]	Cross-Sectional Studies	Large Chinese adolescent cohorts (*n* > 12,000)	Confirmed a robust statistical chain linking early menarche and menstrual/premenstrual distress to elevated depression rates and increased NSSI/suicidal behavior.	Cross-sectional architecture prevents definitive chronological or causal formatting.
Jung et al., 2019 [77]	National Health Survey Analysis	Large representative population data	Examined systemic biological/hormonal correlates and risk factors underpinning depressive trajectories and suicidal behaviors.	Residual confounding from socio-economic or background family factors.
Sequeira et al., 2017 [81]	Mendelian Randomization Study	Large UK genetic sample (*n* > 3000)	Established a causal genetic framework linking early menarcheal timing directly to increased depressive symptoms during early-to-mid adolescence.	Unable to capture real-time, proximal environmental stress fluctuations.
Roberts et al., 2020 [82] (ALSPAC)	Population-Based Birth Cohort	*n* = 3741 British female participants	Definitive long-term evidence showing early menarche elevates self-harm risk from adolescence through emerging adulthood, while late menarche acts as a shield.	Subject to standard attrition/missing data typical of decades-long cohort studies.
Sontag et al., 2011 [84] Miller et al., 2018 [86]	Multi-Wave Prospective Investigations	Targeted adolescent female cohorts	Explores the acute, reciprocal relationship between chronic peer/interpersonal stress, pubertal timing, and the subsequent maintenance of NSSI.	Smaller sample sizes compared to national registries; high reliance on self-reports.
Silva et al., 2017 [85]	Mediation Analysis Study	College student sample	Romantic relationship distress acts as a critical proximal trigger for self-harm, fully or partially mediated by severe deficits in emotion regulation.	Retrospective tracking of adolescent dynamics; limited to individuals entering higher education.
Iron-Segev et al., 2025 [87]	National Youth Survey	Representative adolescent registry (2025 Data)	Confirmed that early menarche is uniquely and cross-sectionally tied to a heightened risk of disordered eating behaviors in girls.	Does not map the longitudinal trajectory of eating disorders into later adulthood.

## Data Availability

No new data were created or analyzed in this study. Data sharing is not applicable to this article.
